# Prevalence of, and Factors Associated With, Symptoms of Anxiety and Depression in Young Adults With a Symptomatic Knee After ACL Reconstruction: Results from the SUPER-Knee study

**DOI:** 10.1177/23259671251371155

**Published:** 2025-09-25

**Authors:** Hilary J.A. Grover, Andrea M. Bruder, Thomas J. West, Kay M. Crossley, Michael A. Girdwood, Jamon L. Couch, Christian J. Barton, Ewa M. Roos, Adam G. Culvenor

**Affiliations:** * Australian College of Sports and Exercise Physicians, Melbourne, Australia; † La Trobe Sport and Exercise Medicine Research Center, School of Allied Health, Human Services and Sport, La Trobe University, Melbourne, Victoria, Australia; ‡ Department of Physiotherapy, Podiatry and Prosthetics and Orthotics, La Trobe University, Melbourne, Victoria, Australia; § Australian IOC Research Centre, La Trobe University, Melbourne, Victoria, Australia; ‖ Department of Sports Science and Clinical Biomechanics, University of Southern Denmark, Odense, Denmark; Investigation performed at La Trobe University, Melbourne, Victoria, Australia

**Keywords:** anterior cruciate ligament, anxiety, depression, knee, mental health, rehabilitation

## Abstract

**Background:**

Anterior cruciate ligament (ACL) injury and its lengthy recovery period can lead to adverse mental health outcomes. However, symptoms of anxiety and depression after ACL injury are infrequently investigated.

**Purpose:**

To investigate the prevalence of, and factors associated with, symptoms of anxiety or depression in young adults with a symptomatic knee after ACL reconstruction (ACLR).

**Study Design:**

Cross-sectional study; Level of evidence, 3.

**Methods:**

We analyzed baseline data from 184 adults with a symptomatic knee (defined as a mean score of <80/100 from 4 subscales of the Knee injury and Osteoarthritis Outcome Score [KOOS_4_]) 9 to 36 months after ACLR who were enrolled in the SUPER-Knee trial (age 30 ± 6 years, 37% women, body mass index [BMI] 27.3 ± 5.2 kg/m^2^). Symptoms of anxiety or depression were assessed using the “anxiety/depression” item of the EQ5D 5-level questionnaire (EQ5D-5L) (1 = none, 2 = slight, 3 = moderate, 4 = severe, and 5 = extreme). Knee-related burden was assessed with the 5 KOOS subscales (0 = worst, 100 = best). The relationship between anxiety or depression with (1) demographic (age, sex, BMI, preinjury activity level, and socioeconomic status) and injury/surgery-related factors (combined meniscal/cartilage injury, >1 ACLR), and (2) KOOS and return to sports (RTS) was evaluated with ordered logistic regression.

**Results:**

A total of 104 (57%) participants reported symptoms of anxiety or depression (slight = 69, moderate = 33, and severe = 2). A higher BMI was the only demographic/injury/surgery-related factor associated with the severity of anxiety or depression symptoms (not anxious/depressed = 26.3 ± 4.5 kg/m^2^, slightly anxious/depressed = 27.7 ± 5.4 kg/m^2^, moderately/severely anxious/depressed = 28.7 ± 6.2 kg/m^2^). More severe anxiety or depression was associated with worse scores on all KOOS subscales, but not RTS.

**Conclusion:**

Over half of adults with knee symptoms 9 to 36 months after ACLR reported anxiety/depression symptoms, and almost 1 in 5 reported moderate to severe anxiety/depression symptoms. Greater severity of anxiety or depression symptoms was associated with overweight/obesity and worse knee-related burden. Mental health outcomes should be monitored after ACLR, and appropriate management and/or referral should be considered.

ANZCTR: ACTRN12620001164987.

Anterior cruciate ligament (ACL) rupture is a common sports-related injury, particularly in athletes involved in jumping, cutting, and pivoting sports.^19^ Regardless of management approach—either surgical (ie, ACL reconstruction [ACLR] + rehabilitation) or nonsurgical (ie, rehabilitation alone)—the rehabilitation process typically exceeds 9 to 12 months, and it is both physically and mentally demanding.^10,11^ Despite returning to sports being a common goal, only two-thirds return to their preinjury level of sport; even fewer (55%) return to the same competitive level of sport.^1^ In the longer term, persistent pain is common,^3^ experienced by up to one-third of individuals 6 years after ACLR^36^; and the development of early-onset knee osteoarthritis occurs within a decade for approximately half of these young adults.^9^

Coming to terms with an ACL injury, its lengthy recovery period, and undesirable long-term outcomes can elicit negative thoughts and feelings—including distress, anger, low self-esteem, anxiety, and even depression.^6^ However, symptoms of anxiety and depression after ACL injury are infrequently investigated. For example, a 2022 systematic review of self-reported symptoms of anxiety and depression after ACL injury found only 2 studies that specifically reported the prevalence of depression.^29^ The highest prevalence (42%) was during the first 6 weeks after ACLR,^16^ dropping to 6% 5 to 20 years after ACL injury.^15^ However, because of the retrospective nature of the studies and small sample sizes, both studies were rated as low quality. More recently, a 2023 United States (USA)-based health care database study of 42,174 patients, found that 11% of patients had a new depression/anxiety diagnosis after ACLR, with women having almost twice the diagnosis rate of men, and those undergoing a secondary ACLR surgery (ie, revision ACLR on the index knee or ACLR on the contralateral knee) also at a higher risk.^8^ However, no studies have evaluated symptoms of anxiety and depression specifically in those with persistent knee pain and difficulties, such as limitations in activities of daily living (ADL) and sports participation, within the first 5 years after ACLR—a time when these previously active and healthy young people are coming to grips with an often-diminished ability to participate in desired activities, occupations, and sports.

Understanding the rates of anxiety and depression symptoms in the large group of individuals who do not return to preinjury status is important, as the presence of anxiety and depression has been associated with inferior outcomes after ACL injury.^38^ A recent systematic review reported that, compared with patients undergoing ACLR without depression, those with a preoperative depression diagnosis were less adherent to postoperative rehabilitation, and reported more pain interference and worse physical function scores within the first postoperative year.^17^ A similar story exists for total knee replacement.^21^

Investigating the prevalence of, and factors associated with, symptoms of anxiety and depression in people with knee symptoms after ACLR could assist in developing strategies to improve ACLR outcomes. Therefore, in young adults with ongoing symptoms 9 to 36 months after ACLR, the present study aimed to (1) investigate the prevalence and severity of self-reported anxiety and depression symptoms, and (2) explore whether the severity of anxiety and depression symptoms are associated with demographic or injury/surgery-related factors, self-reported knee burden, and return to sports (RTS).

## Methods

### Study Design and Participants

This study used baseline data from the 184 participants enrolled in the Supervised Exercise Therapy and Patient Education Rehabilitation (SUPER-Knee) randomized controlled trial.^12^ The trial was completed at a single university site (La Trobe University) in Melbourne, Australia, with detailed methods and recruitment results published.^12,13^ Briefly, from community advertisements and invitation letters sent to consecutive patients who had undergone ACLR at 1 of 12 collaborating private orthopaedic clinics and 9 public hospitals, we enrolled patients between February 2021 and April 2023 who signed written informed consent and met the following inclusion criteria: (1) Patients who underwent ACLR within the last 9 to 36 months—ensuring that participation did not conflict with routine clinical care; (2) those aged 18 to 40 years at the time of ACLR; and (3) those with ongoing symptoms in their ACLR knee—defined as a mean score of <80/100 from 4 subscales of the Knee injury and Osteoarthritis Outcome Score (KOOS_4_) covering pain, symptoms, function in sport/recreation, and quality of life^33^. The exclusion criteria were as follows: (1) concomitant intra-articular fracture; (2) knee reinjury, surgery, or injection in the past 3 months; (3) rehabilitation for the ACLR knee in the last 6 weeks; (4) planned surgery to ACLR knee in the next 12 months; and (5) presence of other health conditions impacting physical function. At the time of enrolment, participants attended La Trobe University for the collection of participant characteristics, anthropometrics, and patient-report outcomes (collected electronically via a secure web-based platform, Research Electronic Data Capture [REDCap]). Ethics approval was granted through La Trobe University (HEC-19447) and Alfred Hospital (HREC 537/19) ethics committees.

### Anxiety and Depression Symptoms

General anxiety or depression symptoms were assessed at the time of enrolment with the EQ5D five-level questionnaire (EQ5D-5L) “anxiety/depression” item. The EQ5D-5L is a valid, reliable, and standard nondisease-specific measure of health status made up of 5 domains (anxiety/depression, pain/discomfort, usual activities, mobility, and self-care), with each domain answered on a 5-point Likert scale reflecting current health status.^30^ The EQ5D-5L anxiety/depression item is scored from 1 to 5 (1 = I am not anxious or depressed; 2 = I am slightly anxious or depressed; 3 = I am moderately anxious or depressed; 4 = I am severely anxious or depressed; and 5 = I am highly anxious or depressed). The EQ5D anxiety/depression item has been used to assess the prevalence of anxiety and depression symptoms in other musculoskeletal studies using a cutoff of “any symptoms” on the EQ5D-3L^34^ (ie, EQ5D-5L levels 2, 3, 4, and 5).

### Demographic and Injury/Surgery-related Factors

Participants were asked to self-report their sex and date of birth (to calculate age), history of ACLR on the contralateral knee, and postcode to quantify socioeconomic status based on the Index of Relative Social Advantage and Disadvantage for the state of Victoria^35^ (median = 50th percentile). Height and weight were measured with a wall-mounted stadiometer and digital scales, respectively, and body mass index (BMI) was calculated. The Tegner Activity Scale, with scores ranging from 0 (sick leave because of knee problems) to 10 (elite level football of any code: Australian football, soccer, and rugby), was used to assess preinjury activity level. Surgical ACLR reports were obtained to determine concomitant injuries/procedures (injury/surgery to meniscus or cartilage), graft type, and whether the procedure was a primary or revision ACLR.

### Knee-related Symptomatic Burden

Knee-related burden was assessed with the KOOS, a 42-item patient-reported outcome measure consisting of 5 subscales: Pain, Symptoms, ADL, Sport and Recreation (Sport/recreation), and Quality of Life (QoL). Participants rated each item on 5 graded adjectival response options; the mean subscale scores were then calculated and converted to a standardized score ranging from 0 to 100, with 100 representing “no problem.” The KOOS is widely used and is valid and reliable in knee-injured populations.^7,34^

### Return to Sports

The RTS status was assessed with a single question: “*Since your ACL surgery, have you returned to your preinjury level of sport (same sport and same level of competition regularly)?”* Participants responded: yes, no, or not applicable (ie, did not play a sport before injury).

### Statistical Analysis

The severity of anxiety or depression symptoms was reported descriptively. Because of the small number of participants with severe anxiety or depression symptoms, when assessing the association with demographic and injury/surgery-related factors, KOOS, and RTS outcomes, we grouped participants with moderate and severe anxiety or depression symptoms together. Unadjusted ordered logistic regression assessed whether the severity of anxiety or depression symptoms was associated with demographic and injury/surgery-related factors, while ordered logistic regression, adjusted for age, sex, and BMI assessed the association between the severity of anxiety or depression symptoms and KOOS and RTS outcomes. Those who did not play competitive sports before ACL injury were excluded from the analysis of RTS outcomes. Statistical analyses were performed with Stata Version 18.0 (StataCorp), with α set at .05.

## Results

The 184 participants enrolled in the SUPER-Knee trial had a mean age of 30 ± 6 years, and two-thirds (63%) were men. Also, 108 (59%) participants had concomitant injuries (combined ACL injury with injury/surgery to meniscus or cartilage), and 40 (22%) had >1 ACLR (either previous ipsilateral or contralateral knee) (Table 1). Most (n = 157; 85%) participants were regularly active in sports in the year before ACL injury (Appendix Table A1), and 167 (91%) were playing sports at the time of ACL injury (Appendix Table A2).

**Table 1:** Participant Baseline Demographic Characteristics*^*a*^*

Baseline Variable	N = 184
Female sex, n (%)	68 (37)
Age, years	30 ± 6
Height, m	1.74 ± 0.09
Weight, kg	82.4 ± 16.5
BMI, kg/m^2^	27.3 ± 5.2
Current smoker, n (%)	21 (11)
Tegner Activity Scale preinjury, median (IQR)	8 (7-9)
Return to preinjury level of sport, n (%)*^*b*^*	22 (14)
Socioeconomic status, median (IQR) percentile	67 (35-84)
>1 previous ACLR, n (%)*^*c*^*	40 (22)
Graft type of most recent index ACLR, n (%)*^*d*^*	
Hamstring tendon	150 (82)
Quadriceps tendon	17 (9)
Bone-patellar tendon-bone	12 (7)
Donor (allograft)	5 (3)
Type of injury to index knee, n (%)*^*e*^*	
Isolated ACL injury	63 (34)
Combined ACL injury with injury/surgery to the meniscus or cartilage	120 (66)
Combined ACL injury with surgery to MCL, LCL, and PCL*^*f*^*	3 (2)
Months between ACL injury and ACLR, median (IQR)	5 (2-10)
Years between ACLR and baseline	2.3 ± 0.7

a Values are presented as mean ± SD, unless otherwise indicated. ACL, anterior cruciate ligament; ACLR, anterior cruciate ligament reconstruction; BMI, body mass index; IQR, interquartile range; LCL, lateral collateral ligament; MCL, medial collateral ligament; PCL, posterior cruciate ligament.

b The denominator is the number of participants playing sports preinjury (n = 157).

c Previous ACLR to either knee.

d Data obtained from surgical records or self-reports when surgical records were unavailable.

e Data obtained from surgical records of 183 participants. Combined injury refers to the presence of a concomitant meniscal tear (either untreated or treated surgically) and/or a cartilage defect of at least grade 2 and/or treated surgically (eg, debridement).

f All 3 participants had concurrent surgery on the MCL.

A total of 104 (57%) participants reported symptoms of general anxiety or depression (slight = 69 [38%]; moderate = 33 [18%]; and severe = 2 [1%]). No demographic or injury/surgery-related factors were associated with the severity of anxiety or depression symptoms, except for the BMI. A higher BMI was associated with more severe anxiety or depression symptoms (Table 2).

**Table 2:** Demographic and Injury/Surgery-related Factors by Severity of Anxiety or Depression Symptoms*^*a*^*

	EQ5D-5L Score for Anxiety or Depression Item			
Variable	None (n = 80)	Slight (n = 69)	Moderate/Severe (n = 35)	*P*
Female sex, n (%)	26 (33)	27 (39)	15 (43)	.248
Age, years, mean ± SD	30 ± 6	30 ± 5	29 ± 5	.598
BMI, kg/m^2^, mean ± SD	26.3 ± 4.5	27.7 ± 5.4	28.7 ± 6.2	**.016**
Socioeconomic status (median, IQR)	64 (33-86)	64 (36-80)	76 (58-86)	.221
>1 previous ACLR, n (%)	16 (20)	16 (23)	8 (23)	.654
Combined injury, n (%)	48 (61)*^*b*^*	47 (68)	25 (71)	.218
Preinjury Tegner Activity Scale score ≥9, n (%)	41 (51)	32 (46)	19 (54)	.992

a The bold *P* value indicates statistical significance. ACLR, anterior cruciate ligament reconstruction; BMI, body mass index; EQ5D5L, EQ5D 5-level questionnaire; IQR, interquartile range.

b The denominator was 79, as combined injury data from the surgical report was missing for 1 participant.

**Figure 1.**
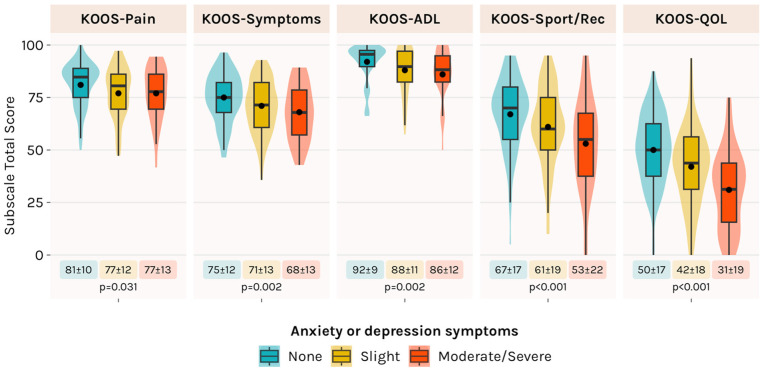
KOOS results based on the severity of anxiety or depression symptoms. Data are presented as mean ± standard deviation. ADL, activities of daily living; KOOS, Knee injury, and Osteoarthritis Outcome Score; QoL, quality of life; Rec, recreation.

Participants with more severe anxiety or depression symptoms reported significantly worse KOOS scores on all subscales (Figure 1). Of the 157 participants who played sports preinjury, 22 (14%) returned to competitive sports after ACLR. The severity of anxiety or depression symptoms was not associated with a lack of return to competitive sports. Return to preinjury sport rates in those with no, slight, and moderate/severe anxiety or depression symptoms were 20%, 14%, and 12%, respectively (*P* = .315).

## Discussion

The main finding in this study was that symptoms of anxiety or depression were present in 57% of young adults aged 18 to 40 years, with ongoing knee symptoms 9 to 36 months after ACLR. The severity of anxiety or depression symptoms was mainly mild, with 18% and 1% reporting moderate and severe anxiety or depression symptoms, respectively. A greater severity of anxiety or depression symptoms was associated with a higher BMI and worse knee-related burden. Clinicians need to be aware of potential mental health impairments and facilitate appropriate support beyond the initial post-ACLR recovery and rehabilitation phases.

### Prevalence of Anxiety or Depression Symptoms

The prevalence of any anxiety or depression symptoms we observed (57%) adds to the limited evidence on mental health outcomes after ACLR, particularly beyond the period of usual postoperative recovery. The rate of anxiety or depression symptoms we observed is slightly higher than the few reports of depression symptoms in the immediate postoperative period (1-6 weeks after ACLR) (42%),^16^ a time when the severity of depression symptoms has been suggested to peak,^29^ and much higher than reported^15^ rates in individuals with knee difficulties 5 to 20 years after ACLR (6%). These previous studies used a definition of depression closely aligned with a clinical diagnosis. Our approach (single EQ5D item) likely classified some individuals as having symptoms of depression not sufficiently severe enough to meet clinical diagnostic thresholds, particularly as most experienced only slight symptoms. Given that the EQ5D item does not differentiate between anxiety or depression symptoms, the high rates we observed in our cohort may also reflect levels of anxiety symptoms rather than depression. Anxiety and depression are also not unique to ACL injury and can affect athletes after a wide variety of musculoskeletal injuries. For example, an Australian Institute of Sport study^20^ evaluating the mental health of athletes found that one-quarter of athletes who were depressed were experiencing a current injury.

Compared with Australian population normative values for the EQ5D-5L anxiety/depression item in those aged 18 to 44 years,^32^ the rates of slight (~30% general population vs 38% current study) and moderate symptoms (~20% general population vs 18% current study) we observed were similar. However, only 1% of our cohort had severe or extreme symptoms, while normative rates for similar-aged adults reporting^32^ severe or extreme anxiety/depression symptoms on the EQ5D-5L were 12% to 22%. While it may be surprising that more severe symptoms of anxiety or depression are not observed in symptomatic young adults after ACLR compared with population norms, patients experiencing such symptoms may have been less likely to volunteer for SUPER-Knee (eg, due to lack of motivation), an exercise-based clinical trial. It is also possible that the anxiety or depression symptoms we observed 9 to 36 months after ACLR were preexisting and not related to the ACL injury or surgery itself. This is unlikely for all participants, as results from an extensive health care database, The Truven database, which comprises over 135 million unique individuals from Marketscan Commercial claims and encounters, and Medicare Supplemental databases, in the USA, indicate that 11% of people experience a new depression/anxiety diagnosis after ACLR.^8^ It is also common for athletes in general to report^31^ depressive symptoms (range 4%-68%). However, a 2017 meta-analysis concluded that athletes were just as likely as nonathletes to report depressive symptoms.^18^ Without preinjury data, we are unable to confirm the rates of incident anxiety or depression symptoms after ACLR in our cohort. Nevertheless, it is essential to screen for anxiety and depression symptoms after ACLR, as adolescents and young adults (a time when ACL injuries typically occur) experience a high rate of mental illness and suicide (the second most common cause of death in this age group),^22^ and suicidal ideations directly related to ACL injury have been reported.^23^

### Being Overweight and Obese Is Associated With Worse Anxiety or Depression Symptoms

Greater BMI was the only demographic factor we evaluated that was associated with the severity of anxiety or depression symptoms. Preinjury demographic factors that we assessed, which were not associated with anxiety or depression, included age, sex, physical activity level, socioeconomic status, and injury/surgery-related factors (>1 ACLR, combined injury). The association between being overweight and obese and anxiety/depression is not new. There is a well-established relationship between BMI and symptoms of depression and anxiety in the general population.^2,14^ Our finding of the relationship between anxiety or depression symptoms and greater BMI after ACLR extends data showing a relationship between BMI and depression symptoms 5 to 20 years after ACLR.^15^ The potential effect of a higher BMI on anxiety and depression symptoms is particularly pertinent in young adults after ACLR, as these individuals are more likely to exhibit elevated BMI relative to uninjured age-, sex-, and activity level-matched controls.^25^ People post-ACLR also tend to progressively put on weight,^24^ particularly central/visceral adiposity,^25^ likely due to the inactivity often imposed by a lengthy rehabilitation period and premature cessation of sport participation. In contrast to our findings, an extensive USA database study of 42,174 ACLRs observed that female sex and second ACLR were independently associated with postoperative depression/anxiety diagnosis.^8^ However, the absolute difference they observed was relatively small (7% between men and women, 4% between reoperation and no reoperation). Whether these differences are clinically relevant remains unclear, and our study may have been underpowered to detect sex differences in the prevalence of anxiety and depression symptoms.

### Anxiety or Depression Symptoms Are Associated With Worse Knee-related Burden

The severity of anxiety or depression symptoms was associated with knee-related burden, with more severe anxiety/depression symptoms corresponding to worse scores on all KOOS subscales. While these data are the first to report on the relationship between anxiety or depression symptoms and knee-related burden in those with ongoing knee symptoms after ACLR, our findings extend results from a recent systematic review showing a link between preoperative depression and inferior outcomes after ACLR.^17^ Because of the cross-sectional nature of our study, we were unable to confirm whether the severity of anxiety/depression was causally linked to poor knee-related outcomes, or vice versa. Nevertheless, our data indicate that the KOOS Sport/Recreation and QoL subscales are most strongly associated with anxiety/depression symptoms. These data may reflect the social and personal status that young adults experience because of their sporting participation and performance. Even though our study showed an association of anxiety/depression symptoms with the KOOS Sport/Recreation subscale, we did not find a significant association between RTS and the severity of anxiety or depression symptoms. Although lower rates of depression symptoms have been associated with increased rates of moderate-to-vigorous physical activity and sports participation,^26,28^ our finding is consistent with other cross-sectional studies on people with knee difficulties after ACLR.^15^ In a 2019 systematic review of psychological factors affecting RTS after ACLR, the authors also observed that only 6% of people cited depression symptoms as the reason for not returning to sports.^27^ Given that anxiety or depression symptoms are implicated in greater knee burden after ACLR, they should be assessed and considered in rehabilitation programs to enable referral to appropriate support and management. The notion of considering mental health outcomes aligns with the recent OPTIKNEE consensus recommendations.^37^

### Limitations

Because of the cross-sectional design of this study, we were unable to confirm whether the anxiety/depression symptoms we observed were preexisting or developed after ACLR. In addition, we could not evaluate the potential for any causal relationship of anxiety/depression with ongoing knee symptoms or BMI. As all participants were enrolled in the parent clinical trial based on the presence of knee symptoms, our results may not be generalizable to individuals without ongoing knee symptoms after ACLR. However, those with ongoing knee symptoms make up a significant portion (one-third) of people after ACLR.^36^ The lack of a comparison group of patients post-ACLR with no/minimal knee symptoms is a limitation. There is also a possibility of selection bias, given that those with more anxiety/depression may have been more likely to enroll in SUPER-Knee for the chance to receive benefits from exercise therapy and education. Another limitation is the use of a single EQ5D-5L item to assess anxiety or depression symptoms. Although other studies used the EQ5D-3L in a similar way,^34^ both the EQ5D-3L and EQ5D-5L items do not differentiate between the 2 related but separate constructs. Although linked, anxiety and depression are individual conditions, and anxiety itself is an umbrella term covering a range of more specific conditions, ranging from generalized anxiety disorder to phobias and stress reactions. Anxiety about returning to sports and ACL reinjury is common after ACLR and is a significant theme in qualitative studies of ACL-injured athletes.^4,5^ The high rates of anxiety or depression symptoms we observed likely also reflect the predefined criteria of our SUPER-Knee cohort requiring a symptomatic knee. The EQ5D-5L is also not used as a clinical tool to measure anxiety or depression. As our study was ancillary to a larger clinical trial, we were limited to using this single item, and the limited eligibility window after ACLR restricted our ability to evaluate the influence of time after ACLR on anxiety/depression symptoms. Finally, we had very few participants reporting severe or extreme symptoms of anxiety or depression, which meant our results reflect associations with mostly mild to moderate symptoms.

## Conclusion

Over half of young adults with knee symptoms 9 to 36 months after ACLR reported anxiety or depression symptoms, and 1 in 5 reported moderate to severe anxiety or depression symptoms. Greater severity of anxiety or depression symptoms was associated with being overweight/obese and having worse knee-related burden. Mental health outcomes should be monitored after ACLR, and appropriate management and/or referral should be considered.

## Appendix

**Appendix Table A1:** Preinjury Sports Activity Characteristics*^*a*^*

Sport	Number (%) of Participants
Soccer	49 (27)
Australian Rules Football	26 (14)
Basketball	26 (14)
Netball	24 (13)
Martial arts	6 (3)
Alpine skiing/snowboarding	3 (2)
Tennis/squash	3 (2)
Dance/aerobics/gymnastics/ballet	3 (2)
Ultimate Frisbee	3 (2)
Cricket	2 (1)
Volleyball	2 (1)
Motorcycle trial/enduro	2 (1)
Rugby	2 (1)
Golf	1 (1)
American Football	1 (1)
Powerlifting	1 (1)
Ice Hockey	1 (1)
Baseball	1 (1)
Roller Derby	1 (1)
Did not participate in sports	27 (15)

a In response to the question: “Did you play sports in the year before your ACL injury?” If yes, what was the primary sport you played?” ACL, anterior cruciate ligament.

**Appendix Table A2:** Sport/Activity Participating in at the Time of ACL Rupture

Sport/Activity	Number (%) of Participants
Sport-related	167 (91)
Soccer	40 (22)
Basketball	26 (14)
Australian Rules Football	25 (14)
Netball	22 (12)
Futsal	15 (8)
Skiing	8 (4)
Martial arts	6 (3)
Ultimate frisbee	3 (2)
Trampoline	2 (1)
Cricket	2 (1)
Cheerleading	2 (1)
Rugby	2 (1)
Running	2 (1)
Snowboarding	2 (1)
Cycling	1 (1)
Gymnastics	1 (1)
Volleyball	1 (1)
Powerlifting	1 (1)
Roller Derby	1 (1)
Baseball	1 (1)
American football	1 (1)
Kickboxing	1 (1)
Tennis/Squash	2 (1)
Non-sport related	17 (9)
Walking/stairs	5 (3)
Dancing	4 (2)
Work-related	3 (2)
Motorbike	2 (1)
Jumping/hopping (not sport related)	2 (1)
Horse riding	1 (1)
